# Socioeconomic Trajectories From Birth to Adolescence and Risk Factors for Noncommunicable Disease: Prospective Analyses

**DOI:** 10.1016/j.jadohealth.2012.06.022

**Published:** 2012-12

**Authors:** Pedro C. Hallal, Valerie L. Clark, Maria Cecilia Assunção, Cora L.P. Araújo, Helen Gonçalves, Ana M.B. Menezes, Fernando C. Barros

**Affiliations:** a Postgraduate Program in Epidemiology, Federal University of Pelotas, Pelotas, Brazil; b Postgraduate Program in Health and Behavior, Catholic University of Pelotas, Pelotas, Brazil

**Keywords:** Socioeconomic factors, Poverty, Adolescent, Chronic diseases

## Abstract

**Purpose:**

To evaluate the associations between family socioeconomic trajectories from 0 to 11 years of age and risk factors for noncommunicable disease at 15 years.

**Methods:**

Individuals born in the city of Pelotas, Brazil, in 1993 are part of a birth cohort study. Socioeconomic position, collected at birth and at 11 years of age, was our main exposure. Risk factors for chronic disease were collected at 15 years. Body mass index was transformed into *Z* score using the World Health Organization standard. Transport and leisure-time physical activity, smoking, and alcohol consumption were assessed by self-report. Blood pressure was measured using a digital sphygmomanometer.

**Results:**

Of 5,249 cohort members, 85.7% were located at the 15-year follow-up visit. Rich adolescents were more likely to be overweight, be obese, and not use active modes of transport to school. Poor adolescents were more likely to smoke. In relation to socioeconomic trajectories, the odds of obesity were 46% higher among those who were “always rich” compared with those who were “always poor”; the odds of use of an inactive mode of transportation were 326% greater among the “always rich” than the “always poor,” whereas the reverse was observed for smoking (odds of 200%). The “always rich” had one-half the odds of walking or cycling to school compared with those who became wealthy in the studied period.

**Conclusions:**

Adolescent socioeconomic position is a stronger determinant of risk factors for noncommunicable diseases than socioeconomic trajectories. However, trajectories do matter, particularly in terms of use of active transportation to school.

**Implications and Contribution**

Rich adolescents were more likely to be overweight, be obese, and use inactive modes of transport to school, but poor adolescents smoked more. Wealthy adolescents who were also wealthy at birth had one-half the odds of walking or cycling to school compared with those who became wealthy in the studied period.

Adult noncommunicable disease is connected with the emergence of risk during childhood and adolescence. In terms of behavioral factors, the risk of adult physical inactivity has been found to be lower for those who were physically active during adolescence [1], whereas adulthood smoking has been linked to childhood smoking experimentation [2]. Risk outcomes such as high blood pressure and overweight or obese status according to body mass index (BMI) during childhood have also been found to be related to adulthood risk outcomes and disease [3,4].

One major determinant of health and well-being worldwide is socioeconomic position (SEP). Both absolute (e.g., income <US $1 per day) and relative (e.g., belonging to the bottom 20% of the income distribution) indicators of poverty have been shown to be related to a series of negative health outcomes [5]. However, most research on this topic relies either on cross-sectional associations between SEP and health outcomes or on longitudinal analysis using SEP at baseline as the exposure variable. Particularly in countries experiencing economic and epidemiological transition, analyses using SEP trajectories are essential for deepening our understanding on the effects of SEP on health.

SEP can be measured in a variety of ways that include both composite and univariate measures [6]. For analyses aiming to examine SEP as an exposure to a given outcome, composite measures of SEP will tend to offer the soundest results. However, conclusions made using composite SEP instruments do not offer clear direction for policy makers. Thus, univariate SEP measures such as income or educational attainment are often used [6] to provide clearer implications for policy.

SEP has a significant impact on adult health, risk factors for and development of disease outcomes. Childhood SEP has been found to have an effect on adulthood morbidity and mortality, independent of adult SEP [7]. When studying the low SEP as a sustained exposure from childhood through adulthood, the cumulative effect leads to a higher risk of coronary heart disease [8]. In terms of behavioral and outcome risk factors, SEP may be associated with physical activity levels [9–11], smoking status [10,12], alcohol consumption [11], and body composition [11].

Studies have reported various outcomes based on changes in SEP over time. In the 1982 Pelotas (Brazil) Birth Cohort Study, Barros et al showed that SEP in early life was more important than SEP in adolescence for determining attained height, whereas the opposite was true for weight [13]. In a birth cohort study from South Africa, results generally indicated that SEP at birth was more strongly associated with lean mass index, whereas SEP at age 9/10 years was more closely related to fat mass index [14]. In the Dutch GLOBE study, Giskes et al observed that whereas childhood SEP was a stronger determinant of BMI in women, adulthood SEP was a stronger determinant among men [15].

Using data from the Coronary Artery Risk Development in Young Adults (CARDIA) study in the United States, researchers found that declines in income tended to be associated with the incidence of hypertension [16]. Melchior et al observed that lifelong poverty was associated with an increased risk of premature mortality in both men and women [17]. In the Framingham Offspring Study from the United States, cumulative poverty was associated with the incidence of type 2 diabetes in women, but not in men [18].

The aim of the present study was to evaluate the prospective associations between family SEP trajectories from 0 to 11 years of age and risk factors and outcomes related to noncommunicable disease (namely, physical activity, smoking and alcohol consumption, and BMI and blood pressure) at 15 years in a birth cohort study from Brazil.

## Methods

### Study population

Individuals born in the city of Pelotas, Brazil, in 1993 are part of an ongoing birth cohort study. Of 5,265 newborns in that calendar year, 5,249 were enrolled. Mothers were interviewed some hours after delivery. Family income in the previous month was reported. Newborns had their weight and length measured by our research team. Pelotas is a medium-sized city in the extreme south of Brazil, near the border with Uruguay. Although belonging to the south macro region of the country, which is one of the wealthiest and most developed ones, Pelotas is less developed than the average of the south region. Extreme income inequalities are observed in the city, so that, for example, the median family income (US $150) is three times lower than the mean family income (US $450). In 2004–2005, we conducted a follow-up visit for all the participants of the cohort. We were able to locate 4,452 participants, who represent 87.5% of the cohort, taking into account that 141 subjects had died. Family income in the previous month was again recorded.

### SEP measurement

Family income at birth and at age 11 years was initially noted as the sum of the salaries of all household members in the previous month, expressed in the Brazilian currency (1 Brazilian real = ∼2 US dollars), thus generating continuous variables. The variables were then divided into tertiles. Therefore, socioeconomic trajectories from birth to 11 years of age had nine possible categories: lowest tertile in both visits (n = 890); lowest tertile at birth and intermediate tertile at 11 years (n = 696); lowest tertile at birth and highest tertile at 11 years (n = 274); intermediate tertile at birth and lowest tertile at 11 years (n = 368); intermediate tertile in both visits (n = 466); intermediate tertile at birth and highest tertile at 11 years (n = 397); highest tertile at birth and lowest tertile at 11 years (n = 162); highest tertile at birth and intermediate tertile at 11 years (n = 277); and highest tertile in both visits (n = 755). To make differences more evident, in some analyses, we contrasted only those who were at the bottom tertile in both periods (n = 890) with those who were in the upper tertile in both periods (n = 755). SEP at 11, instead of 15, years was used to evaluate the effect of previous socioeconomic change on current behavior at 15 years of age. Otherwise, socioeconomic change would be too recent, with insufficient time to influence behavior. However, analyses using SEP trajectories from 0 to 15 years were run, and results were comparable with those obtained using SEP change from 0 to 11 years.

### Risk outcomes

Outcome variables were collected at 15 years of age when we were able to locate 4,325 cohort members. Weight and height were measured twice using standardized equipment, and the mean value was used. BMI was transformed into *Z* score using the World Health Organization standard. Overweight was defined as a BMI *Z* score >1 and <2, whereas obesity was defined as a BMI *Z* score ≥2.

Blood pressure was measured twice using a digital sphygmomanometer. Blood pressure measurements followed routine protocols [19], including rest for at least 15 minutes and seated position. Systolic and diastolic blood pressure mean values were analyzed as continuous variables, with correction equations based on a validation study [19]. Smoking and alcohol consumption in the preceding month were assessed by self-report.

### Risk behaviors

Transport and leisure-time physical activity (LTPA) were measured using a validated questionnaire [20]. The questionnaire initially asked about the primary mode of transportation to and from school. Those who reported going to school by car, bus, or motorcycle were categorized as using “inactive” modes of transportation. Those reporting walking or cycling to school were classified as “active” in transport. A list of several leisure-time physical activities, including sports, was also presented, and participants were asked whether they had practiced those activities during the past week. For each positive answer, information on weekly frequency and duration was obtained. Frequency was multiplied by time to create a weekly score for each activity; these scores were then added up, generating an LTPA score. We classified adolescents according to whether they reached 300 min/wk of LTPA [21].

For smoking, we categorized adolescents as “no” if reporting 0 days and “yes” if reporting 1 or more days. With regard to alcohol consumption, we categorized adolescents as “never drinkers” (0 d/m), “less than once a week” (1–4 d/m), and “weekly or more frequently” (≥5 d/m).

### Data analyses

We initially described the sample in terms of risk factors for chronic disease at 15 years. Then, we presented the proportion of adolescents in each category of the outcome variables by the nine possible SEP trajectories and the mean (standard deviation) systolic and diastolic blood pressure in the same categories without any adjustment for confounders. Next, we ran adjusted analyses for each outcome variable either by logistic regression (all categorical outcomes) or by linear regression (blood pressure). These analyses were adjusted for birth weight, maternal schooling at birth, birth order, pubertal status (measured using the Tanner stages at 15 years of age), and sex. In the blood pressure analyses, height was also adjusted for. We then analyzed SEP at 15 years only. Finally, we contrasted those who were consistently poor against those who were consistently rich using the *χ*^2^ test.

### Ethics

All phases of the 1993 Pelotas (Brazil) Birth Cohort Study obtained institutional review board approval. Written informed consent was provided by parents or guardians in all visits, and verbal consent was given by adolescents at the 11- and 15-year follow-up visits. Further details about the 1993 Pelotas (Brazil) Birth Cohort Study are available elsewhere [22].

## Results

In previous publications, we showed that subjects lost to follow-up were comparable with those located in terms of sociodemographic factors and behavioral variables in early life [23]. For example, the average family income at birth was just 3% higher among those lost to follow-up as compared with those located in adolescence.

Table 1 describes our sample in terms of risk factors for chronic disease at 15 years of age. Prevalence of obesity was 10.2% among boys and 7.2% among girls. Most participants (73.4%) reported an active transportation mode to school. Boys were more active than girls in leisure time and were less likely to report smoking. Weekly or more frequent alcohol consumption was reported by 4.7% of the adolescents. Mean systolic pressure was higher among boys than girls, whereas the opposite was observed for diastolic pressure.

In Table 2, the unadjusted analyses of socioeconomic trajectories and risk factors for chronic disease are presented. Regardless of baseline SEP, there was a trend of increasing prevalence of obesity from the poor to the rich group in adolescence. With regard to active transportation to school, rich subjects were consistently less likely to use active transportation. However, baseline SEP was related to the outcome; among those who were poor and became rich, 74% walked or cycled to school; among those who were consistently rich, only 47% walked or cycled to school. Smoking was strongly related to adolescent poverty, regardless of baseline SEP. The other variables were not clearly associated with SEP trajectories in the unadjusted analyses. The adjusted analyses (Table 3) confirmed these findings in regard to smoking and active transportation; however, obesity was no longer significantly associated with SEP trajectories.

**Figure 1:**
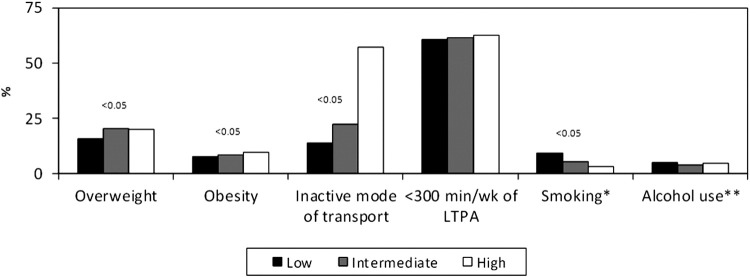
Risk factors for chronic disease at 15 years of age according to socioeconomic tertiles at 11 years. SEP = socioeconomic position; LTPA = leisure-time physical activity. * At least one cigarette in the previous month; ** weekly or more frequent alcohol intake.

Figure 1 presents the associations between SEP at 11 years of age and risk factors for chronic diseases at age 15 years. Rich adolescents were significantly more likely to be overweight or obese and use a passive mode of transport to school, but were significantly less likely to smoke. LTPA and alcohol consumption were not significantly related to adolescent SEP.

**Figure 2:**
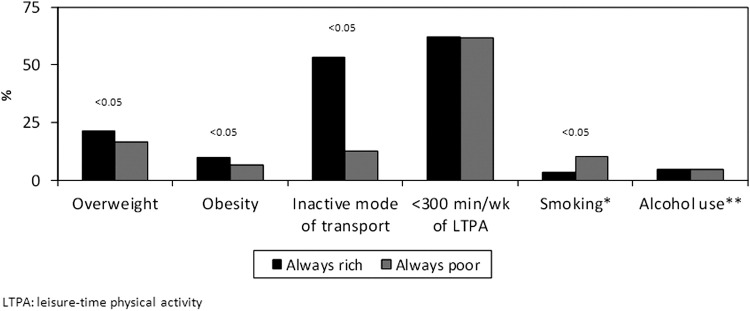
Inequalities in risk factors for chronic disease at 15 years of age: comparison between the extreme groups. Always rich: families belonging to the top tertile of income at birth and at 11 years of age. Always poor: families belonging to the bottom tertile of income at birth and at 11 years of age. * At least one cigarette in the previous month; ** weekly or more frequent alcohol intake.

Figure 2 compares two groups of adolescents: those who were born “always poor” and those who were “always rich.” The latter were more likely to be overweight, be obese, and use an inactive mode of transportation to school, whereas “always-poor” adolescents were more likely to smoke. Achieving 300 min/wk of LTPA and alcohol consumption were similar in these two extreme groups. Sex differences were evident in the comparison of these two extreme groups in terms of obesity. The odds of being obese were 143% higher in “always-rich” boys than “always-poor” adolescents, whereas no significant differences were observed among girls.

## Discussion

In this prospective study from Brazil, we found SEP to be an important determinant of several risk factors for chronic disease. In addition, we found that SEP trajectories do matter, particularly in terms of transport-related physical activity. To the best of our knowledge, this finding was never reported before.

Few studies are available on the influence of SEP trajectories on health outcomes. Consistent with our finding that BMI trends upward in adolescents experiencing improvements in SEP, Griffiths et al found that South African adolescents in a high SEP category at age 9/10 years were at risk for having an increased fat mass index [14]. This finding is consistent with ours and with the Developmental Origins of Health and Disease literature, indicating that fatness is programmed at a later stage of life as compared with lean mass [24,25]. In our older cohort including individuals born in 1982, it was clear that height is determined in early life, whereas weight is influenced by factors operating afterward [13].

Methodologically, some research has attempted to examine SEP trajectories using retrospective data to establish childhood SEP [15,17,24]. The prospective nature of our study avoids recall bias in the estimates of early-life SEP. Some limitations of our study should be taken into account. With regard to measurement, we do not have data on body composition or accelerometry-based physical activity for the full cohort. Also, we relied on self-reported smoking and alcohol consumption. These methods have limitations; however, the impact of those limitations on the associations described here is unlikely to be major. In addition, these biases are more likely to affect measures of occurrence (as those described in Table 1) than measures of effect (as those described in all other tables and figures). We opted to use family SEP indicators instead of adolescents' own SEP because most did not have any income in the previous month and most are also still in school. Strengths of our study include the population-based nature of the cohort, with a wide range of SEP, as well as the high follow-up rates.

Giskes et al found that childhood SEP was an important predictor for adulthood BMI in women but reported that adulthood SEP was more closely related to differences in BMI among men. Women in this study who reported having been in a low SEP during childhood according to paternal occupation were more likely to be obese or overweight at follow-up. However, men in low SEP during adulthood were more likely to have an increased BMI. We opted not to stratify our main analyses based on nine SEP trajectories by sex owing to sample size limitations. However, in the comparison among the two extreme groups (“always poor” vs. “always rich”), we found that the higher odds of obesity among the better-off are observed in boys, but not among girls. In sensitivity analyses, we reran our analyses using the nine groups separately for boys and girls, and none of the interaction tests were statistically significant.

With regard to physical activity, Cleland et al found that, in Australia, upward social mobility from 9 to 36 years was related to increased physical activity and fitness levels [9]. In contrast, our study found no significant difference in LTPA levels according to any SEP indicator, even in the comparison between “always-rich” and “always-poor” individuals. However, we found a significant effect of SEP trajectories on commuting physical activity. First, we reproduced the well-established association between commuting physical activity and poverty in Brazil [26–28], which is observed both for children (e.g., commuting to school) and adults (e.g., commuting to work). More interestingly, however, we found that wealthy adolescents who were also wealthy at birth had one-half the odds of walking or cycling to school compared with those who became wealthy in the studied period. This finding has important public health implications. There is evidence that physical activity habits acquired early in life tend to track into adulthood [29,30]. Therefore, it is possible that those adolescents who were used to walking or cycling to school were less likely to adopt a passive mode of transportation after becoming rich, as compared with those who were consistently rich and used to going to school by car. However, car possession explains part of our findings. In our cohort, car ownership was reported by 79% of the “always rich” and by 56% of those who became rich in the 11-year period.

Brazil is undergoing a markedly positive economic transition. Health and socioeconomic inequalities have declined over recent years [31]. Because of economic growth, cash transfer programs, and increased levels of the minimum wage, poverty is being rapidly reduced. The impact of this rapid economic transition on health is still unknown. Evidence suggests that maternal and child health outcomes related directly to poverty are likely to improve with the country's economic development [32]. On the other hand, our data suggest that challenges in terms of noncommunicable disease are likely to arise from this transition [33]. Our findings are derived from a single city in the south of the country and, therefore, are not immediately applicable to the whole country's population. However, the fact that Brazil is experiencing rapid economic development in recent years suggests that the effect of these rapid development on noncommunicable disease risk should not be ignored. Policy makers, public health experts, and health professionals, who deal directly with patients, will urgently need to find alternatives to prevent chronic diseases among poor families who become less poor rapidly. Otherwise, the already high burden of noncommunicable disease in Brazil is likely to get even higher in the coming years.

## Figures and Tables

**Table 1:** Description of the sample in terms of risk factors for chronic disease at 15 years of age

Risk factors for chronic diseases	All	Boys	Girls	*p*
Body mass index (*Z* score)				.001
Normal (>1)	72.4%	70.4%	74.4%	
Overweight (1–1.99)	18.9%	19.4%	18.5%	
Obesity (≥2)	8.7%	10.2%	7.2%	
Active mode of transport to school	73.4%	77.2%	69.8%	<.001
≥300 min/wk of LTPA	38.1%	54.0%	23.0%	<.001
Smoking	6.0%	4.8%	7.1%	.001
Alcohol consumption				.003
Never	75.1%	77.4%	73.0%	
Less than once a week	20.2%	18.1%	22.2%	
Weekly or more frequently	4.7%	4.5%	4.8%	
Systolic blood pressure (mean ± SD)	119.4 (9.8)	123.3 (10.3)	115.5 (10.4)	<.001
Diastolic blood pressure (mean ± SD)	67.6 (6.2)	66.4 (4.7)	68.9 (7.2)	<.001

LTPA = leisure-time physical activity; SD = standard deviation.

**Table 2:** Prevalence of risk factors for chronic disease at 15 years of age according to socioeconomic trajectories from birth to 11 years. Unadjusted analyses

Risk factors for chronic diseases	Socioeconomic trajectories from birth to 11 years									*p*
	Low-low	Low-int	Low-high	Int-low	Int-int	Int-high	High-low	High-int	High-high	
Overweight	15.7%	19.0%	16.1%	14.2%	20.0%	21.6%	20.0%	25.3%	21.4%	.007
Obesity	6.7%	8.0%	10.0%	9.1%	8.9%	10.0%	8.0%	9.5%	9.8%	
Inactive mode of transportation	12.5%	19.7%	26.6%	16.3%	21.5%	32.9%	17.2%	30.1%	53.2%	<.001
<300 min/wk of LTPA	61.7%	62.9%	58.2%	60.5%	59.2%	65.3%	57.3%	62.0%	62.3%	.57
Smoking	10.2%	6.4%	2.7%	8.4%	4.5%	3.2%	8.4%	4.6%	3.4%	<.001
Weekly or more frequent alcohol use	4.8%	4.2%	5.0%	4.4%	4.4%	4.5%	5.9%	3.5%	4.6%	.16
Systolic blood pressure (mean ± SD)	119 (10)	120 (9)	120 (10)	120 (10)	119 (9)	120 (10)	119 (9)	119 (9)	119 (10)	.39
Diastolic blood pressure (mean ± SD)	67 (7)	68 (6)	68 (6)	68 (6)	68 (6)	68 (6)	68 (6)	68 (6)	67 (6)	.11

Int = intermediate socioeconomic tertile.

**Table 3:** Odds ratios and linear regression coefficients for risk factors for chronic disease at 15 years of age according to socioeconomic trajectories from birth to 11 years. Adjusted analyses

Risk factors for chronic diseases	Socioeconomic trajectories from birth to 11 years									*p*
	Low-low	Low-int	Low-high	Int-low	Int-int	Int-high	High-low	High-int	High-high	
Obesity	.8 (.5, 1.4)	.7 (.5, 1.4)	1.0 (.7, 1.8)	1.2 (.7, 2.0)	.9 (.6, 1.5)	.8 (.5, 1.4)	.8 (.4;1.7)	1.0 (.6;1.7)	1.0	.65
Inactive mode of transportation	.3 (.2, .4)	.4 (.3, .5)	.5 (.3, .7)	.3 (.2, .5)	.4 (.3, .5)	.5 (.4, .7)	.3 (.2, .4)	.5 (.4, .8)	1.0	<.001
<300 min/wk of LTPA	.9 (.6, 1.2)	.9 (.7, 1.3)	.8 (.6, 1.2)	.8 (.6, 1.1)	.8 (.6, 1.0)	1.0 (.7, 1.4)	.6 (.4, .9)	1.0 (.7, 1.4)	1.0	.26
Smoking	1.9 (1.1, 3.6)	1.1 (.6, 2.1)	.5 (.2, 1.4)	1.7 (.9, 3.0)	1.0 (.5, 2.0)	.6 (.3, 1.5)	1.5 (.7, 3.6)	.7 (0.3, 1.8)	1.0	.007
Weekly or more frequent alcohol use	.8 (.4, 1.5)	.6 (.3, 1.1)	.9 (.4, 2.0)	.7 (.3, 1.5)	.9 (.5, 1.7)	.9 (.5, 1.7)	1.2 (.5, 2.9)	.5 (.2, 1.3)	1.0	.70
Systolic blood pressure	−1.2 (−2.4, −.1)	.0 (−1.1, 1.1)	.5 (−.9, 2.0)	−.2 (−1.6, 1.1)	.1 (−1.3, 1.3)	.0 (−1.2, 1.2)	.8 (−.9, 2.5)	.1 (−1.3, 1,4)	.0	.15
Diastolic blood pressure	−.6 (−1.5, .2)	.0 (−.8, .9)	.2 (−.8, 1.2)	.1 (−.9, 1.0)	.3 (−.6, 1.1)	.4 (−.4, 1.3)	.6 (−1.6, 1.9)	.0 (−1.0, 1.0)	.0	.33

Values are odds ratios (outcomes 1–6) or regression coefficients (outcomes 7–8). Adjusted for sex, maternal schooling, birth weight, birth order, and pubertal status (analyses were also adjusted for height when the outcome was blood pressure).
